# What Turns Assistive into Restorative Brain-Machine Interfaces?

**DOI:** 10.3389/fnins.2016.00456

**Published:** 2016-10-13

**Authors:** Alireza Gharabaghi

**Affiliations:** Division of Functional and Restorative Neurosurgery, and Centre for Integrative Neuroscience, Eberhard Karls University TuebingenTuebingen, Germany

**Keywords:** assistive technology, neurorehabilitation, stroke, rehabilitation robotics, brain-computer interface, brain-robot interface

## Abstract

Brain-machine interfaces (BMI) may support motor impaired patients during activities of daily living by controlling external devices such as prostheses (assistive BMI). Moreover, BMIs are applied in conjunction with robotic orthoses for rehabilitation of lost motor function via neurofeedback training (restorative BMI). Using assistive BMI in a rehabilitation context does not automatically turn them into restorative devices. This perspective article suggests key features of restorative BMI and provides the supporting evidence: In summary, BMI may be referred to as restorative tools when demonstrating subsequently (i) operant learning and progressive evolution of specific brain states/dynamics, (ii) correlated modulations of functional networks related to the therapeutic goal, (iii) subsequent improvement in a specific task, and (iv) an explicit correlation between the modulated brain dynamics and the achieved behavioral gains. Such findings would provide the rationale for translating BMI-based interventions into clinical settings for reinforcement learning and motor rehabilitation following stroke.

In stroke patients with severe and persistent motor deficits, restitution of useful function is very limited. Despite intensive rehabilitation programs, these patients are often left with a dysfunctional upper extremity and, consequently, with a long-term dependency on others for activities of daily living (Jørgensen et al., 1999; Dobkin, 2004; Feigin et al., 2008). There are many technology-driven efforts to improve recovery in this patient group on the basis of motor re-learning. Brain-machine interfaces (BMI), for example, have been applied lately to bridge the impaired connection in the sensorimotor loop. Unlike classical BMIs that *assist* motor impaired patients, for example by controlling external devices such as prostheses, their *restorative* counterparts provide brain-state dependent proprioceptive feedback by way of orthotic devices attached to the hand or arm of the patient to facilitate rehabilitation training toward functional restoration. Such supported movements facilitate the detection of motor intention even in the absence of actual movements (Gomez-Rodriguez et al., 2011; Brauchle et al., 2015). When used in conjunction with commercially available robotic rehabilitation technology (Bauer et al., 2015; Brauchle et al., 2015; Vukelić and Gharabaghi, 2015a), these devices are also known as brain-robot interfaces (BRI). Patient control over these robotic training devices is usually mediated by motor imagery-related sensorimotor oscillations of the ipsilesional cortical electroencephalogram (EEG) or electrocorticogram (Gharabaghi et al., 2014b). However, the translation from assistive toward restorative BMI cannot be realized simply by applying the identical method in altered environments for different goals. We, therefore, propose a conceptual framework for restorative BMI distinct from their assistive predecessors, summarize the most recent supporting evidence (Kraus et al., 2016a; Naros et al., 2016), and project future developments and perspectives in this field. We exemplify this concept for the area of movement recovery after stroke. The underlying assumption is that learning to modulate sensorimotor beta-oscillations might facilitate post-stroke functional restoration. Current evidence for this assumption is circumstantial: (i) Movement-related beta-oscillations are compromised in stroke patients and correlate with the impairment level (Rossiter et al., 2014). (ii) Volitional modulation of sensorimotor beta-oscillations can be learned via BMI and correlates with corticospinal excitability increases (Kraus et al., 2016a) and motor learning (Naros et al., 2016) in healthy subjects. However, there is currently only one pilot study available in literature that has addressed this concept of “learning beta-band self-regulation” for post-stroke rehabilitation (Naros and Gharabaghi, 2015). This perspective article intends to outline in detail the rationale for this approach and to initiate a discussion on necessary features and prerequisites of restorative BMI for stroke rehabilitation.

## From assistance to restoration

Despite the application of novel techniques such as BMI training combined with physiotherapy (for an overview, see Naros and Gharabaghi, 2015), there is still a lack of relevant functional improvement of the hand and finger function in the patient group with persistent deficits of the upper limb. This has attracted interest in the specificity and mechanisms of BMI therapy, since the underlying neurophysiology of this intervention (Kraus et al., 2016a), together with appropriate strategies to optimize learning and motor gains (Naros et al., 2016) have not yet been fully explored.

The BMI approach in rehabilitation comes into play once standard physical practice is no longer possible in the targeted patient group. Consequently, the lost motor function following stroke limits the re-learning of movements (Doyon and Benali, 2005; Halsband and Lange, 2006; Naros et al., 2016). In such cases, motor imagery (MI) might constitute an alternative for physical practice (Halsband and Lange, 2006; Boe et al., 2014) since it activates the sensorimotor system without any overt movement (Gao et al., 2011; Szameitat et al., 2012; Vukelić and Gharabaghi, 2015a; Naros et al., 2016). This volitional modulation of oscillatory activity during MI can be supported by providing BMI feedback about the user's current brain state to facilitate operant learning of oscillatory patterns considered beneficial to recovery (Vukelić and Gharabaghi, 2015a; Naros et al., 2016). The concept of restorative BMI training, therefore, is based on the premise that associative learning facilitates self-regulation of such MI-related brain activity by connecting the neural correlates of movement intention and the contingent feedback. Notably, for behavioral gains, this approach requires not only operant learning of brain self-regulation but also the progression of the trained brain dynamics (see also paragraph on Adaptive BMI feedback). More specifically, movement-related desynchronization (ERD) in the contralateral, ipsilesional sensorimotor cortex is compromised in stroke patients compared to healthy controls, i.e., the more severe the patient's motor impairment, the less ERD (Rossiter et al., 2014). Accordingly, a restorative training approach would need to increase this oscillatory modulation range again.

However, most BMI studies do not demonstrate such a progressive modulation range, i.e., the improved skill of brain self-regulation in the course of the training, even when behavioral gains are reported after the intervention (for an overview, see Naros et al., 2016). This suggests that the reported BMI use had general priming effects on subsequent physiotherapy rather than genuine effects, i.e., intervention specific motor gains (for an overview, see Naros et al., 2016). The demonstration of direct brain-function and/or brain-behavior relationships would, however, be a requirement for the concept of restorative BMI, which is based on the assumption that operant conditioning of the targeted brain state and dynamics facilitates task-specific motor gains (Naros and Gharabaghi, 2015). Unlike previous ambivalent findings during BMI *motor* rehabilitation, neurofeedback-induced operant conditioning of the targeted brain states was indeed successfully achieved in the *cognitive* domain and led to task-specific functional gains (Zoefel et al., 2011). This ambiguity between the findings in the cognitive and the motor domain might be related to the methodological limitations of earlier BMI approaches in the motor domain. This applies in particular to the cortical frequency-band trained by ERD in previous studies, i.e., alpha- instead of beta-oscillations, the feedback strategy and/or the application or lack of additional brain stimulation (Naros and Gharabaghi, 2015; Naros et al., 2016).

## Adaptive BMI feedback

Classical BMI approaches maximize the classification accuracy of the device to optimally detect task-related MI (Thomas et al., 2013; Thompson et al., 2013; Spüler et al., 2014; Bauer and Gharabaghi, 2015a). This approach has been applied in most previous studies with stroke patients without resulting in a gain of the skill for BMI control as one might have expected in a continuous learning experience (for an overview, see Naros and Gharabaghi, 2015). On the basis of learning principles, a certain degree of challenge for the participant, therefore, seems to be required to reinforce continuous effort and progression of brain self-regulation (Bauer and Gharabaghi, 2015a). In this context, mathematical simulations (Bauer and Gharabaghi, 2015b) and empirical data (Naros and Gharabaghi, 2015; Naros et al., 2016) suggest that dynamic threshold adaptation of the oscillatory desynchronization level that has to be achieved to control the BMI in the course of the training, i.e., adjusting the difficulty level of the neurofeedback task on the basis of the performance, is a more appropriate method for achieving BMI reinforcement learning than a fixed threshold at maximum classification accuracy, i.e., an unchanged oscillatory desynchronization level that has to be achieved to control the BMI (Theodoridis and Koutroumbas, 2009).

Along these lines, a recent study with healthy subjects over a 3-day training period was the first to demonstrate that dynamic threshold adaptation is instrumental in facilitating learning of movement-related brain self-regulation. By contrast, subjects who trained with a classical BMI concept, i.e., without threshold adaptation, failed to progressively modulate the targeted brain activity (Naros et al., 2016). This matched the concept that BMI paradigms which focus on the maximization of classification accuracy optimize the metabolic cost (Jackson and Fetz, 2011; Naros et al., 2016). Restorative BMIs should, therefore, be designed in such a manner as to provide incentives not only for achieving but also for enhancing the targeted brain activity, e.g., progressing the level of ERD (Carmena, 2013; Naros and Gharabaghi, 2015; Naros et al., 2016). Although, BMI tasks are potentially linked to the experience of frustration (Fels et al., 2015), a less demanding task structure of reaching the brain state only once so as to be rewarded with feedback did not result in improved brain self-regulation (Naros et al., 2016). By contrast, the more demanding task of providing or withholding feedback contingent to the targeted brain self-regulation was essential for achieving sustained ERD (Naros et al., 2016). More specifically, when comparing different BMI training conditions in a parallel-group design [(i) adaptive classifier thresholding and contingent feedback, (ii) adaptive classifier thresholding and non-contingent feedback (iii) non-adaptive classifier thresholding and contingent feedback, (iv) non-adaptive classifier thresholding and non-contingent feedback], contingent neurofeedback and adaptive classifier thresholding were critical for learning brain self-regulation which, in turn, led to behavioral gains after the intervention. Contingent feedback to successful brain self-regulation meant that as soon as the predefined ERD level was achieved the participants were rewarded by the robotic opening of the hand. However, if the targeted brain state could not be sustained, the robotic movement ceased again but could be resumed within the same trial if the predefined brain state was attained again (Naros et al., 2016). Furthermore, adaptive classifier thresholding throughout the intervention was realized by adjustments of the task difficulty before each training session in the course of a multi-session program. These adjustments were made in accordance with the BMI performance in the preceding session based on an algorithm that has been shown to support reinforcement learning of self-regulated beta-oscillations (Naros and Gharabaghi, 2015).

In this context, future studies may evaluate the impact of different task thresholds, i.e., targeted ERD levels, on the learning incentive, thereby empirically determining the optimal difficulty level for brain self-regulation and disentangling the relative contribution of neurofeedback specificity and sensitivity (Bauer et al., 2016a). Moreover, future approaches may investigate alternative approaches to balancing the mental effort involved, for example by adjusting the task demands on the basis of self-ratings by the participants (Bauer et al., 2016b).

## Brain-function interaction

While the progression of sensorimotor self-regulation is a necessary requirement for restorative BMI, such local modulation would not be sufficient by itself. Such an intervention would necessitate more global network effects as well to bring about behavioral gains. But how would BMI feedback training translate self-regulated modulation of local oscillations into changes of distant functional networks? In other words, how is such a brain-function interaction physiologically mediated?

Imaging studies based on multi-channel electroencephalography revealed that sensorimotor brain self-regulation and BMI feedback entrained an extended cortical motor network that includes frontal and parietal brain areas (Vukelić et al., 2014; Vukelić and Gharabaghi, 2015a) with distributed, but spatially selective frequency-specific effects on cortico-cortical connectivity that last beyond the intervention period (Vukelić and Gharabaghi, 2015b). This motor network modulation is critically linked to the proprioceptive feedback provided by the BMI (Vukelić and Gharabaghi, 2015a). Notably, those subjects who were particularly capable of performing sensorimotor brain self-regulation could be predicted by a distributed alpha-band resting state network measured before the intervention (Bauer et al., 2015). Similarly, the resting state functional connectivity of the motor cortex seems to be related to motor learning (Mottaz et al., 2015) and to the prediction of functional improvement after stroke (Nicolo et al., 2015). Moreover, functional coupling of coherent theta-band oscillations during the BMI task correlated with the skill of sensorimotor modulation, thus indicating a motor learning-related network (Vukelić and Gharabaghi, 2015a). These findings match well with the neurophysiological concepts that link these various frequency domains to working memory and sensorimotor integration (Fell et al., 2011; Cruikshank et al., 2012), sensory processing and multi-modal integration (Palva and Palva, 2007; Weisz et al., 2014), and the retrieval of stored motor schemata and bottom-up integration of sensory and motor information (Caplan et al., 2003; Cruikshank et al., 2012; Vukelić and Gharabaghi, 2015a).

With regard to the intended behavioral improvements, the modulation of corticospinal connectivity by BMI feedback may represent the even more important functional network effect. Neurofeedback interventions have already been shown to increase the effective corticospinal connectivity, i.e., the sensorimotor excitability evaluated by transcranial magnetic stimulation (TMS) and motor evoked potentials (MEP) (Pichiorri et al., 2011; Shindo et al., 2011; Mokienko et al., 2013). However, until very recently, these measurements did not provide a specific link between the modulated brain activity and the changed connectivity to the periphery. Methodological improvements with refined TMS maps (Kraus and Gharabaghi, 2015, 2016) closed this gap by demonstrating robust changes of corticospinal connectivity for the BMI-trained muscle, but not for the control muscle. The largest MEP gains were found in those cortical areas that were most strongly modulated by the intervention (Kraus et al., 2016a). Furthermore, this target selectivity and topographic specificity were paralleled by a functional correlation between the modulated brain activity and the increased connectivity to the periphery, i.e., the largest MEP gains were observed in the subjects with the biggest modulation range (Kraus et al., 2016a).

Future studies are required to evaluate whether these functional network changes of corticocortical and corticospinal connectivity during the intervention and in the following resting state persist during behavioral tasks after the intervention, and to what extent they influence the respective performance. Furthermore, different feedback modalities, such as functional electrical stimulation and/or closed-loop TMS (Gharabaghi et al., 2014a; Raco et al., 2016; Royter and Gharabaghi, 2016), may be explored in conjunction with BMI technology to compare their differential impact on network modulations with that one of proprioceptive feedback provided by the robotic orthoses in earlier studies. It will be particularly important to explore these brain-function interactions for the whole upper extremity so as to translate them to activities of daily living. This might also entail studying the impact of brain-machine interfaces connected to multi-joint exoskeletons (Grimm and Gharabaghi, 2016; Grimm et al., 2016a,b) for three-dimensional reach-to-grasp movements (Brauchle et al., 2015) on the corticospinal excitability of different muscle groups, including synergetic and antagonistic interactions.

## Brain-behavior interaction

Unlike in the cognitive domain (Zoefel et al., 2011), a brain-behavior interaction in the motor domain, i.e., a direct link between the brain state/dynamics modulated by BMI feedback and subsequent improvements in an actual motor task, was not demonstrated until very recently. In addition to the factors mentioned in earlier paragraphs, the targeted brain state might be one of the major reasons for this lack; this has already been outlined in detail elsewhere and resonates here (Naros et al., 2016): Despite the eligibility of beta-ERD as a control signal in brain interfaces (Bai et al., 2008) the majority of BMI studies up to now preferred to use alpha-ERD (Naros and Gharabaghi, 2015). This was due to the fact that, in stroke patients, alpha-ERD was more effective than beta-ERD in classifying brain states related to movement (Gomez-Rodriguez et al., 2011). Although, these two frequency bands are modulated by motor execution and MI in much the same way (Van Wijk et al., 2012; Kilavik et al., 2013; Brinkman et al., 2014), it is becoming clearer that they perform different tasks. The function of alpha-ERD is to gate the inhibition of regions which are irrelevant for the task (Pineda, 2005; Mazaheri and Jensen, 2010; Sabate et al., 2011), whereas beta-ERD is responsible for mediating sensorimotor cortex disinhibition (Siegel et al., 2012; Kilavik et al., 2013) and muscular proprioceptive feedback (Salmelin et al., 1995; Mima et al., 2000; Riddle and Baker, 2006; Kristeva et al., 2007; Aumann and Prut, 2014). On the basis of these differences in function, we postulated that, of the two frequency bands, beta-oscillations constitute the better therapeutic option for BMI therapy in patients suffering from motor impairment following stroke (Brauchle et al., 2015; Naros and Gharabaghi, 2015). This approach thus allowed the first demonstration of a frequency-specific correlation between the modulation of cortical physiology with MI-based BMI training and later motor performance (Naros et al., 2016). Such a correlation was, however, not observed between alpha-activity and motor performance. Promoting the ability to voluntarily control beta-oscillations on the basis of proprioceptive feedback might, therefore, facilitate the communication between the motor cortex and muscles in the same frequency band (Brown, 2007; Darvishi et al., under review), thereby resulting in improved motor control in behavioral tasks (Naros et al., 2016).

The next step will be to draw a direct comparison between the operant conditioning of different frequency bands, for example between alpha- and beta-band ERD, to ascertain which particular oscillatory pattern is responsible for this improvement. Further interventions may also be required to gain maximal exploitation and consolidation of the patients' remaining ability for motor learning and brain self-regulation. One such additional input during robot-assisted training may be activity-dependent brain stimulation (Gharabaghi, 2015; Massie et al., 2015). During BMI training, for example, concurrent state-dependent transcranial magnetic stimulation is capable of unmasking latent corticospinal connectivity following stroke (Gharabaghi et al., 2014a). On the basis of Hebbian-like plasticity, state-dependent stimulation synchronized to maximum ERD may serve to stabilize the corticospinal circuits involved (Kraus et al., 2016b).

Future studies will explore whether the behavioral gains resulting from beta-ERD modification also lend themselves to other motor tasks. This would be instrumental in transforming such an approach into a clinical application. Some patients may, however, be unable to gain volitional control of this technique using beta-modulation in a standard EEG-based setting on account of an extended cortical lesion and/or distorted physiology. In such instances, the detection and neurofeedback training may be accomplished by epidural recordings of field potentials (Gharabaghi et al., 2014b). This alternative approach, which is nearer to the neural signal source, may not only require a shorter period of therapy to induce clinical gains than is customary using the standard EEG technique (Gharabaghi et al., 2014c), but may also act as a bi-directional interface for concurrent brain stimulation (Gharabaghi et al., 2014d).

In summary, BMIs may be referred to as restorative tools when demonstrating subsequently (i) operant learning and progressive evolution of specific brain states/dynamics, (ii) correlated modulations of functional networks related to the therapeutic goal, (iii) subsequent improvement in a specific task, and (iv) an explicit correlation between the modulated brain dynamics and the achieved behavioral gains. Such findings would provide the rationale for translating BMI-based interventions into clinical settings for reinforcement learning and motor rehabilitation following stroke.

## Author contributions

The author confirms being the sole contributor of this work and approved it for publication.

### Conflict of interest statement

The author declares that the research was conducted in the absence of any commercial or financial relationships that could be construed as a potential conflict of interest.
